# Age-standardized Incidence Rates for Leukemia Associated with Consanguineous Marriages in 68 Countries, an Ecological Study

**DOI:** 10.4084/MJHID.2015.027

**Published:** 2015-04-22

**Authors:** Mostafa Saadat

**Affiliations:** Department of Biology, College of Sciences, Shiraz University, Shiraz 71454, Iran

## Abstract

Consanguineous marriage that defines as a union between biologically related persons has a variety of known deleterious correlations with factors that affect public health within human populations. To investigate the association between the mean of inbreeding coefficient (α) and incidence of leukemia, the present ecological study on 68 countries was carried out. Statistical analysis showed that the age-standardized incidence rate of leukemia positively correlated with log_10_GNI per capita (r=0.699, df=66, P<0.001) and negatively correlated with log_10α_ (r=−0.609, df=66, P<0.001). Controlling log_10_GNI per capita, a significant negative correlation between log_10α_ and the age-standardized incidence rate of leukemia was observed (r=−0.392, df=65, P=0.001). The countries were stratified according to their annual GNI per capita, low and high-income countries with GNI per capita less than and more than 10,000$, respectively. Statistical analysis showed that in high-income countries, after controlling for log_10_GNI per capita, the correlation between the age-standardized incidence rate of leukemia and log_10α_ was still significant (r=−0.600, df=36, P<0.001). It should be noted that there was no significant association between the age-standardized mortality rate due to leukemia and log_10α_ (P>0.05). The present finding indicates that the rate of leukemia, age-standardized for incidence, is lower in countries with a high prevalence of consanguineous marriages.

## Introduction

Consanguineous marriage defined as a union between biologically related persons has a longstanding social habit among some populations. Its prevalence depends on many factors, such as demographic, religious, cultural and socio-economic factors.^1^–^4^

Consanguinity has a variety of known deleterious correlations with factors that affect public health within human populations.^1^,^5^–^14^ However, there were some reports that described a negative association between consanguinity and risk of diseases.^15^–^17^ A negative association was reported between the susceptibility to infection with HIV-1 and inbreeding coefficient.^17^ Based on an ecologic study the mean of inbreeding coefficient (at the population level; α) is negatively associated with age-standardized mortality rate due to breast cancer. This means that the countries with a high level of consanguinity show a low level of age-standardized mortality rates due to breast cancer.^15^ A significant relationship between parental consanguinity and clinical response to chemotherapy among locally advanced breast cancer patients has been reported.^16^

It is well established that genetic components involved in the risk of several types of cancers, including leukemia.^18^–^24^ On the other hand, consanguinity increases the homozygosity of the offspring. Therefore, for countries such as our country, where the consanguineous marriage is common,^25^ the association between consanguinity and incidence of leukemia or mortality due to leukemia is highly important for public health programs. Therefore, the present ecological study was carried out.

## Materials and Methods

### Data collection

The age-standardized rate is the number of new cases or deaths per 100,000 persons per year. An age-standardized rate is the rate that a population would have if it had a standard age structure. Standardization is necessary when comparing several populations that differ with respect to age because age has a powerful influence on the risk of cancer. Data about age-standardized incidence rates and age-standardized mortality rates for leukemia (per 100,000 persons per year) were obtained from the WHO website.^26^ The inbreeding coefficient is the probability that an individual has received both alleles of a pair from an identical ancestral. The mean of inbreeding coefficient (α) values for the countries were obtained from the website.^27^ In the present study, we used gross national income per capita (GNI, annual; at international dollars) as a confounding factor. Data about GNI per capita for 2012 were obtained from the WHO website.^28^ Inclusion criteria for selection of countries were based on the availability of the variables mentioned above (Table 1).

### Statistical analysis

Kolmogorov-Smirnov test indicates that the GNI per capita and α have an abnormal distribution (For GNI per capita: Kolmogorov-Smirnov Z-test=1.276, P=0.077; For α Kolmogorov-Smirnov Z-test=2.043, P<0.001). Logarithmic transformation (log_10_) was used on GNI per capita (named log_10_GNI) and α (named log_10α_), because they had skewed distributions, and the logarithmic transformations brought them closer to a normal distribution. Correlations between the variables having normal distribution or the logarithmic transformation of the variables not showing normal distribution were determined using the parametric Pearson’s correlation coefficient analysis. Multiple regression analysis was carried out. Also, the partial correlation coefficient analysis was carried out. Statistical analysis was performed using SPSS (version 11.5) statistical software package. A probability of P<0.05 was considered statistically significant. All statistical tests were two-sided.

## Results

Table 1 represent the mean of inbreeding coefficients, GNI per capita; age-standardized incidence rate and age-standardized mortality rate in the study countries

There was no significant association between the age-standardized mortality rate due to leukemia and log_10α_ before and/or after controlling for the log_10_GNI per capita (Table 2). However, it is not the same as a previous report that indicates that the age-standardized mortality rate due to breast cancer is negatively correlated with the mean of inbreeding coefficient (α).^15^

Statistical analysis showed that the age-standardized incidence of leukemia positively correlated with the log_10_GNI per capita (r=0.699, df=66, P<0.001) and negatively correlated with the log_10α_ (r=−0.609, df=66, P<0.001). In multiple regression analysis, age-standardized incidence or age-standardized mortality from leukemia were used as dependent variables and log_10_GNI and log_10α_ were used as independent variables. It should be noted that logand log_10α_GNI per capita had a significant correlation with each other (r=−0.531, df=66, P<0.001). However, the multiple regression analysis showed that there was no significant collinearity. Partial correlation analysis was carried out in order to eliminate the effect of possible confounding effect of GNI per capita on the association between the age-standardized incidence rate of leukemia and log_10α_. After checking the log_10_GNI pern capita, the log_10α_ showed a significant negative correlation with the age-standardized incidence of leukemia (r=−0.392, df=65, P=0.001).

We noted a possible lack of reliable data from low-income countries. Therefore, we stratified the countries according to their GNI per capita, low and high-income countries with GNI per capita less than and more than 10,000$, respectively. Statistical analysis showed that in high-income countries, after controlling the log_10_GNI per capita, the correlation between the age-standardized incidence rate of leukemia and log_10α_ was significant (r=−0.600, df=36, P<0.001).

## Discussion

The main finding of the present study is the negative association between age-standardized incidence rate for leukemia and mean of inbreeding coefficient (α). This means that countries are having a high frequency of consanguinity, show a low level of age-standardized incidence rates for leukemia. This finding is not easy to interpret.

A significant positive association between level of inbreeding coefficient (due to consanguineous marriages) and risk of cancers (including leukemia) has been reported by some studies.^29^–^32^ However, several studies had shown that countries with high consanguinity demonstrate lower age-standardized mortality rates and incidence in breast cancer,.^15^,^33^,^34^

It is well established that the prevalence of consanguinity is mostly present in some regions.^1^,^25^,^27^,^35^,^36^ The prevalence of consanguineous marriages is remarkably higher in many Asian and African countries compared with the Western countries. On the hand, the data published by WHO,^26^ show the estimated age-standardized incidence of leukemia in the Asian (3.9 per 100,000 persons per year) and African (3.0 per 100,000 persons per year) countries was significantly lower than in North America (8.7 per 100,000 persons per year) and European countries (7.0 per 100,000 persons per year). At present, it is very difficult to establish how much the consanguinity, high in Eastern countries, contributes to this difference or if this reflects only ethnic and environmental factors.

We know that there are several types of leukemia and genetic elements involved in the pathogenesis of each type might be differing from the other types.^18^–^24^ However, for estimating the age-standardized incidence and mortality rates, all of leukemia types were pooled.

The present study is an ecological study. Other studies (such as case-control and cohort studies) are necessary for concluding that a large proportion of death could attribute to inbreeding due, in several countries, to the high prevalence of consanguinity.

## Figures and Tables

**Table 1:** Mean of inbreeding coefficients, GNI per capita, age-standardized incidence rate and age-standardized mortality rate in the study countries.

Country	GNI per capita	α (10^−4^)	Age standardized incidence rate (per 100,000 persons per year)	Age standardized mortality rate (per 100,000 persons per year)
Afghanistan	1560	332	2.3	2.3
Algeria	8360	152	4.7	3.8
Argentina	11730	3	5.1	3.5
Australia	43300	1	9.4	3.5
Bahrain	18910	165	4.9	2.0
Bangladesh	2030	45	1.5	1.4
Belgium	39860	3	8.3	4.0
Bolivia	4880	3	3.3	2.9
Brazil	11530	21	4.3	3.5
Burkina Faso	1490	355	1.1	1.1
Cambodia	2330	35	5.1	4.8
Canada	42530	4	9.5	3.6
Chile	21310	6	4.9	3.1
China	9040	27	4.3	3.6
Colombia	9990	12	5.8	4.1
Costa Rica	12500	11	5.0	4.1
Croatia	20200	1	6.3	3.7
Czech Re	24720	1	5.9	3.4
Ecuador	9490	13	6.5	5.0
Egypt	6450	94	5.9	5.1
El Salvador	6720	14	5.8	4.7
France	36720	2	8.0	3.7
Guinea	970	131	1.4	1.3
Honduras	3880	11	4.8	4.3
Hungary	20710	1	6.8	4.0
India	3910	238	2.8	2.3
Indonesia	4730	95	4.3	3.7
Iran	10250	185	5.8	4.6
Iraq	4230	225	6.8	6.5
Ireland	35670	1	9.4	3.4
Italy	32920	4	7.7	3.9
Japan	36300	13	4.3	2.7
Jordan	5980	200	6.1	5.1
Kuwait	47770	205	6.5	3.5
Lebanon	14160	91	7.0	5.1
Libya	17430	209	5.1	3.7
Malaysia	16270	47	4.7	4.0
Mexico	16450	1	5.6	3.7
Mongolia	5020	63	1.5	1.1
Morocco	5060	89	3.0	3.5
Netherland	43510	1	6.9	3.4
Nigeria	2450	242	1.5	1.4
Norway	66960	2	7.8	2.9
Oman	25320	169	5.0	4.3
Pakistan	2880	282	2.8	2.6
Panama	15150	6	5.6	3.8
Peru	10090	16	4.7	3.6
Philippine	4380	3	4.7	3.9
Portugal	24770	9	6.8	3.2
Qatar	80470	271	4.9	3.4
Saudi Arabia	30160	223	3.8	2.8
Singapore	60110	20	6.0	3.0
Slovakia	24770	1	8.6	3.8
South Africa	11010	16	3.3	3.0
Spain	31670	6	6.5	3.0
Sri Lank	6030	92	4.7	3.7
Sudan	2070	197	4.5	4.3
Sweden	43980	3	7.1	3.2
Syrian Arabia	5120	236	6.4	5.7
Tanzania	1560	236	1.2	1.1
Tunisia	9210	213	4.0	3.0
Turkey	18190	74	5.8	4.6
UAE	41550	223	3.7	2.3
UK	37340	2	7.5	3.2
Uruguay	15310	9	5.8	4.0
USA	52610	1	8.6	4.2
Venezuela	12920	7	4.0	3.0
Yemen	2310	215	5.6	5.3

**Table 2:** Correlation coefficients between age-standardized rates (ASR) for incidence of leukemia and mortality due to leukemia and study variables.

Variables	df	ASR for incidence		ASR for mortality	
		r	P	r	P
**Log_10_GNI per capita**	66	0.699	<0.001	0.142	0.246
**Log_10_**α	66	−0.609	<0.001	−0.099	0.420
**Log_10_**α*	65	−0.392	0.001	−0.028	0.819
**Log_10_**α**	36	−0.600	<0.001	−0.069	0.682

* After controlling for Log_10_GNI per capita

** After controlling for Log_10_GNI per capita in countries having GNI per capita at least 10,000 $
